# Pharmacological modulation of ferroptosis as a therapeutic target for liver fibrosis

**DOI:** 10.3389/fphar.2022.1071844

**Published:** 2023-01-10

**Authors:** Le Li, Zhijun Zhu

**Affiliations:** ^1^ Liver Transplantation Center, Clinical Research Center for Pediatric Liver Transplantation, National Clinical Research Center for Digestive Diseases, Beijing Friendship Hospital, Capital Medical University, Beijing, China; ^2^ Department of hepatobiliary surgery, Chifeng Municipal Hospital, Chifeng, China

**Keywords:** liver fibrosis, liver transplantation, ferroptosis, ferroptosis inducer, treatment, bioactive compounds

## Abstract

Liver fibrosis, which is characterized by the excessive deposition of extracellular matrix (ECM) materials (primarily fibrillar collagen-I), is an abnormal repair reaction and pathological outcome of chronic liver diseases caused by alcohol abuse, non-alcoholic fatty liver disease, and chronic hepatitis B and C virus infections. Liver fibrosis often progresses to liver cirrhosis and hepatocellular carcinoma. Ferroptosis, characterized by lipid peroxidation, is a form of iron-dependent non-apoptotic cell death, and recent studies have reported that ferroptosis contribute to the development of liver fibrosis. Moreover, several agents have demonstrated therapeutic effects in experimental liver fibrosis models by inducing hepatic stellate cell (HSCs) ferroptosis. This review delineates the specific mechanism by which ferroptosis contributes to the development of liver fibrosis. Specifically, we focused on the different types of therapeutic agents that can induce HSCs ferroptosis and summarize their pharmacological effectiveness for liver fibrosis treatment. We suggest that HSCs ferroptosis may be a potential useful target of novel therapies for preventing and treating liver fibrosis.

## Introduction

Liver fibrosis, characterized by diffuse excessive and progressive deposition of the extracellular matrix (ECM) in liver, is a pathological state and abnormal repair reaction to chronic liver injury that is typically caused by long-standing liver damage resulting from infectious (hepatitis B and C viruses, HBV and HCV), metabolic (non-alcoholic fatty liver disease, NAFLD), toxin- or drug-induced, cholestatic, or autoimmune insults (Friedman and Pinzani, 2022). Continued liver fibrosis exponentially increases the risk of liver-related mortality regardless of the cause of liver disease. Liver fibrosis is one of the most important manifestations of chronic liver diseases and is typically implicated in the progression of disease to cirrhosis, which is associated with severe morbidity and mortality as well as higher incidence of hepatocellular carcinoma (Roehlen et al., 2020). Liver fibrosis has become one of the leading global causes of liver disease and is poised to become a leading indication for liver transplantation. What’s more, liver fibrosis can negatively affect the outcomes and prognosis of both adult and pediatric patients with liver transplantation and lead to retransplantation (Berumen et al., 2021). The molecular pathogenetic mechanism of liver fibrosis has not yet been fully elucidated, and to date, no effective anti-fibrotic drugs have become available to treat liver fibrosis (Gilgenkrantz et al., 2021). Therefore, it is of fundamental importance to delineate the molecular mechanisms underlying the development of liver fibrosis to develop effective management and treatment strategies.

In recent years, ferroptosis, iron-dependent non-apoptotic cell death characterized by lipid peroxidation, has been quickly gaining attention in chronic liver disease research due to the predisposition of the liver to oxidative stress injury and excessive iron accumulation (Wu et al., 2021). Accumulating evidence has demonstrated that ferroptosis plays an important role in the pathogenesis of liver fibrosis (Kim et al., 2020; Wu et al., 2021; Pan et al., 2021; Zhou et al., 2022; Chen et al., 2022). Some bioactive compounds have been demonstrated to exert therapeutic effects in experimental liver fibrosis models *via* pharmacological targeting by inducing HSCs ferroptosis or inhibiting hepatocytes ferroptosis. In the current review, we first delineated the specific mechanism by which ferroptosis contributes to the development of liver fibrosis. Specifically, we focused on the different types of therapeutic agents that can target HSCs or hepatocyte ferroptosis and summarize their pharmacological effectiveness for liver fibrosis treatment. This review is expected to improve our knowledge of the molecular mechanisms of ferroptosis in liver fibrosis and highlight strategies for targeting HSCs or hepatocyte ferroptosis as potential novel therapeutic targets.

## Pathophysiology of liver fibrosis

**FIGURE 1:**
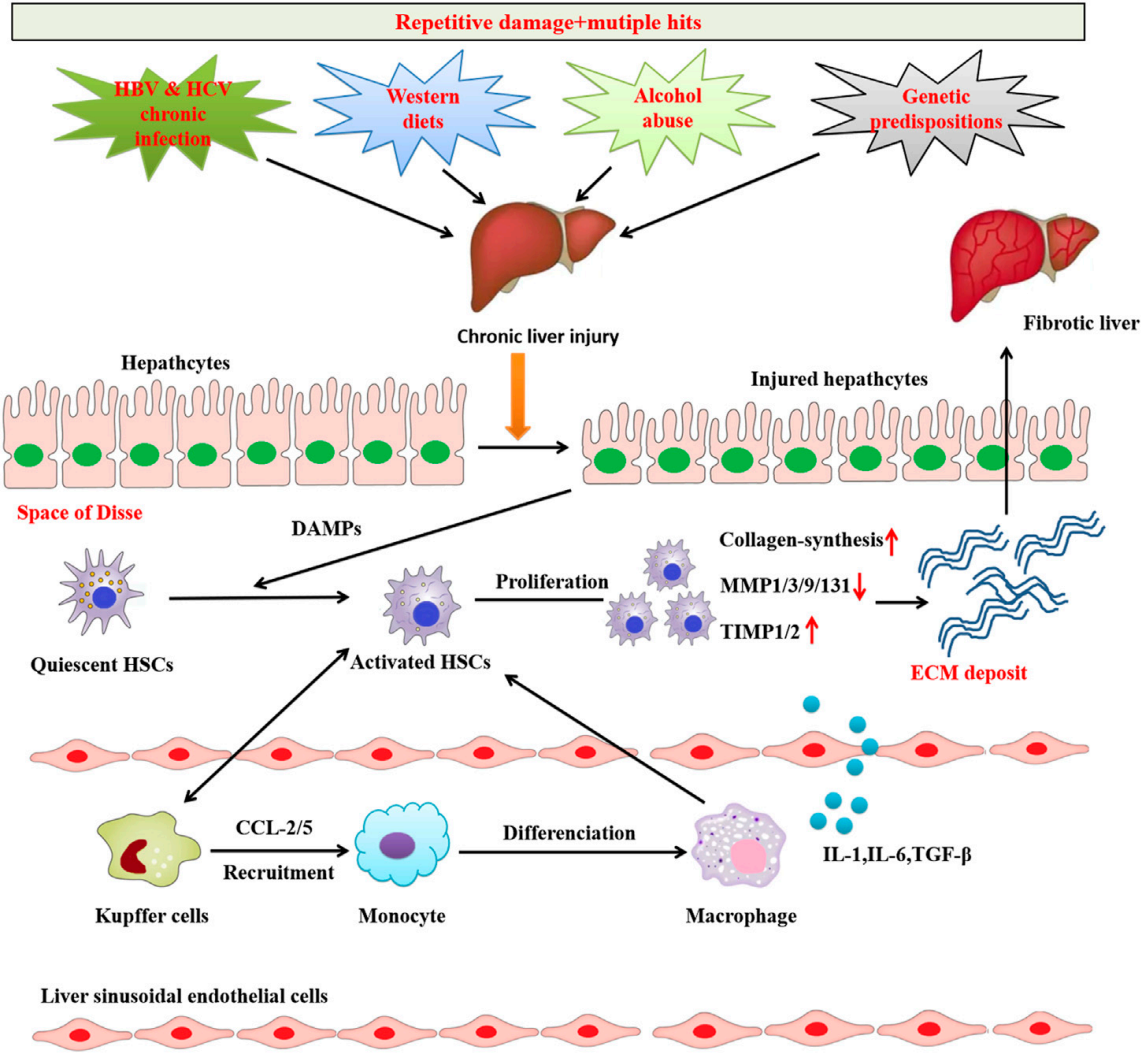
Common mechanisms of liver fibrosis. Repetitive damage and mutiple hits causes chronic hepatocyte injury, which causes release of damage-associated patterns (DAMPs) that activate Hepatic stellate cells (HSCs) and recruit immune cells. Activated HSCs are continuously activated and proliferated to secret abundant fibrogenic cytokines and produce excessive ECM, which causes the imbalance of pro-fibrosis/anti-fibrosismechanism.The pro-fibrosis mechanism leads to the abnormal formation of scar and eventually induces liver fibrosis.

Liver fibrosis leads to the accumulation of ECM proteins, mainly Type I and Type III collagens, to form fibrous scar tissues that replace damaged normal tissue and ultimately compromise normal liver function (Friedman, 2003; Kisseleva and Brenner, 2021). Generally, two types of chronic liver injuries, i.e., hepatotoxic and cholestatic injury, cause liver fibrosis (Kisseleva and Brenner, 2021). Hepatotoxic injury is triggered by chronic cellular injury of hepatocytes from external factors, including HBV and HCV infections, alcoholic steatohepatitis (ASH), NAFLD, and non-alcoholic steatohepatitis (NASH) (Bataller and Brenner, 2005). Primary and/or secondary diseases such as primary biliary cholangitis, primary sclerosing cholangitis, and biliary atresia obstruct or reduce bile flow in the liver, thereby cause cholestatic injury (Bataller and Brenner, 2005; Kisseleva and Brenner, 2021; Friedman and Pinzani, 2022). Regardless of cause, liver fibrosis cases share a common molecular mechanism involving epithelial or endothelial barrier disruption, hepatocyte injury and death, chronic inflammation, and HSCs activation (Dhar et al., 2020) (Figure 1).

### Hepatic stellate cells activation

The activation of HSCs is recognized a core event in liver fibrosis. Under normal conditions, HSCs containing retinoid lipid droplets are located at the space of Disse and show a quiescent phenotype. Quiescent HSCs function as major vitamin A-storing pericytes (Geerts, 2001; Iredale, 2007; Senoo et al., 2007), which are essential for maintaining the quiescent HSCs phenotype (Hazra et al., 2004; She et al., 2005). Chronic liver injury destroys the hepatocyte membrane and leads to hepatocyte necrosis and apoptosis, thereby causing hepatocyte injury. The injured hepatocyte releases damage-associated molecular patterns (DAMPs), which directly stimulate the transformation from quiescent HSCs to activated HSCs. During activation, quiescent HSCs decrease the expression of glial fibrillar acidic protein, vitamin A, and peroxisome proliferator-activated receptor gamma (PPARγ) and lose lipid droplets. Activated HSCs are equivalent to myofibroblasts that express fibrogenic genes such collagen Type I and alpha-smooth muscle actin (α-SMA) (Fallowfield et al., 2007), eventually leading to liver fibrosis (Gandhi, 2017; Schuppan et al., 2018). The imbalance between ECM synthesis and degradation causes excessive ECM accumulation and deposition in the space of Disse and the formation of hepatic scar tissue (Lackner and Tiniakos, 2019). ECM synthesis and degradation is regulated by the balance between matrix metalloproteinases (MMPs) and tissue inhibitors of metalloproteinases (TIMPs). Changes to the MMP/TIMP balance between ECM protein synthesis and degradation can lead to ECM deposition, resulting in the development of hepatic fibrosis. TIMPs inhibit MMPs that promote ECM degradation (Tacke and Weiskirchen, 2012). The activated HSCs acquire contractile capability, upregulate α-SMA expression, and secrete cytokines including transforming growth factor beta 1 (TGF-β1), platelet derived growth factor (PDGF), and connective tissue growth factor (CTGF) (Tan et al., 2021). The chemokines secreted by activated HSCs accumulate chemotactically, thereby perpetuating and aggravating inflammation and fibrogenesis in the inflammatory compartment. DAMPs are also involved in stimulating the activation of Kupffer cells and other immune cells. These cells further stimulate the activation of HSCs and maintain their survival by secreting pro-inflammatory and pro-fibrotic factors such as TGF-β1, PDGF, interleukin-1 beta (IL-1β), and tumor necrosis factor alpha (TNF-α) to induce inflammation (Tan et al., 2021).

### Hepatocytes

Injury and death to hepatocytes that comprise 80% of the total liver cell population are important initial events in all liver disease etiologies. Dead hepatocytes release DAMPs, which play a vital role in the development of fibrosis and inflammation by transducing danger signals to HSCs and Kupffer cells (Berumen et al., 2021; Wang et al., 2022). In response to injury, hepatocytes alter their gene expression profiles and produce several newly expressed fibrogenic factors, such as osteopontin, TGF-β, NADPH oxidase 4 (NOX4), transcription regulator TAZ (WWTR1), and Indian Hedgehog and Notch (Xie et al., 2013; Lan et al., 2015; Wang et al., 2016; Zhu et al., 2018). Moreover, injured hepatocytes can secrete exosomes containing non-coding RNAs (ncRNAs) that regulate HSCs activation (Lee et al., 2017; Liu et al., 2019; Luo et al., 2021a; Luo et al., 2021b; Luan et al., 2022; Ma et al., 2022). Therefore, developing therapies to protect hepatocytes against damage is considered crucial in treating liver fibrosis.

### Inflammatory cells

Studies have increasingly shown that chronic inflammation is a detrimental and critical element of liver fibrosis, as hepatocyte-derived fibrogenic factors alone cannot directly activate HSCs. Many studies have suggested that inflammatory cells such as neutrophils (Gao and Bataller, 2011; Moles et al., 2014; Koyama et al., 2017; Mridha et al., 2017; Gehrke et al., 2018), resident macrophages of liver Kupffer cells (Hellerbrand et al., 1999; Bonniaud et al., 2005; de Gouville et al., 2005), Th17 cells, and bone marrow-derived monocytes (Meng et al., 2012) can promote HSCs activation by releasing cytokines and growth factors. For more detail regarding the role of inflammatory cells and cytokines in liver fibrosis pathogenesis, see previous reviews (Roehlen et al., 2020; Berumen et al., 2021; Kisseleva and Brenner, 2021).

## Molecular mechanisms of ferroptosis

**FIGURE 2:**
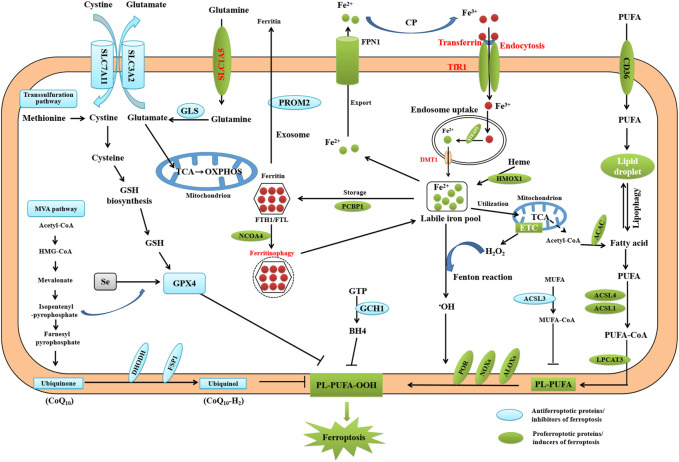
Core mechanisms of ferroptosis. Ferroptosis is mainly caused by iron-dependent lipid peroxidation. The initiation of ferroptosis requires two key signals, namely the accumulation of free iron and the inhibition of antioxidant SLC7A11/GPX4 system. The generation of polyunsaturated phospholipid (by ACSL4 and LPCAT3) and subsequent activation of ALOX have a main role in promoting lipid peroxidation. This process requires HO from an iron-mediated Fenton reaction or the activation of POR, NOX, or mitochondria electron transport chain pathways. The lipid peroxidation or its secondary products (e.g., 4-HNE and MDA) induce pore formation in plasma or organelle membrane, which eventually triggers cell death at the final step of ferroptosis. Alternatively, CoQ10 or tetrahydrobiopterin (BH4) inhibits ferroptosis independently of GSH. Importantly, all aspects of iron metabolism, including iron absorption, storage, export, and utilization, have an important regulatory effect on ferroptosis.

The concept of ferroptosis was first reported by the Stockwell lab in 2012 based on three major research areas, which proposed a foundational understanding of ferroptosis: the mechanisms of lipid and amino acid metabolism (EAGLE, 1955; Mitchell et al., 1973; Bannai et al., 1977), the control of reactive oxygen species (ROS) (Ursini et al., 1982; Flohé, 2020), and the regulation of iron (Stockwell, 2022) (Figure 2). Ferroptosis, which is characterized by the iron-dependent oxidative modification of phospholipid membranes, is a non-apoptotic mode of regulated cell death (RCD) (Dixon et al., 2012). Ferroptosis reflects a delicate imbalance between inducers of ferroptosis and ferroptosis defense systems. When ferroptosis-promoting factors, including iron-dependent ROS and lipid peroxitation (LPO), significantly override the antioxidant defense systems, lipid peroxides lethally accumulate on cellular membranes, which induces membrane rupture and ultimately results in ferroptotic cell death (Lei et al., 2022). Ferroptosis is mainly driven by iron accumulation and LPO causing subsequent plasma membrane rupture (Tang and Kroemer, 2020). The induction of ferroptosis requires two key signals: the accumulation of free iron and the inhibition of the antioxidant SLC7A11-GSH-GPX4 system (Chen et al., 2021). Ferroptosis defense systems include the SLC7A11-reduced glutathione (GSH)-glutathione peroxidase 4 (GPX4), dihydroorotate dehydrogenase -dihydroubiquione (DHODH-CoQH2), ferroptosis suppressor protein 1-ubiquinol (FSP1-CoQH2), and GTP cyclohydrolase 1-tetrahydrobiopterin (GCH1-BH4) systems (Lei et al., 2022). Primary substrates for LPO are phospholipid polyunsaturated fatty acids (PL-PUFAs) due to their intrinsic susceptibility to peroxidation chemistry (Hadian and Stockwell, 2020). PL-PUFAs are generated by different enzymes such as acyl-coenzyme A [CoA] synthetase long-chain family member 4 (ACSL4) and lysophosphatidylcholine acyltransferase (LPCATs), which incorporate free PUFAs into phospholipids (PL). In non-enzymatic LPO, ACSL4 ligate PUFAs with coenzyme A (CoA) to produce acyl-CoA, which can be re-esterifed in phospholipids through LPCATs to produce PL-PUFAs. Acetyl CoA serve as building blocks for PUFAs synthesis, through the action of acetyl CoA carboxylase (ACC) (Hadian and Stockwell, 2020). Once PL-PUFAs are incorporated into membrane environments, arachidonate lipoxygenase (ALOXs) and cytochrome P450 oxidoreductase (POR), and labile iron use molecular oxygen (O_2_) to do a peroxidation reaction, resulting in the generation of peroxidated PL-PUFAs(PL-PUFA-OOH) (Hadian and Stockwell, 2020; Zou et al., 2020). This process requires hydrogen peroxide (H_2_O_2_) derived from an iron-dependent Fenton reaction or POR and NADPH oxidase (NOX) activation, or mitochondria electron transport chain pathways. During the last step of ferroptosis, LPO or its secondary products such as 4-HNE and MDA cause pore formation in plasma or organelle membranes, which triggers cell death. In recent years, ferroptosis has garnered substantial attention in liver fibrosis research, and there has been an increasing focus on targeting ferroptosis in potential treatment strategies (Wu et al., 2021; Pan et al., 2021).

## Ferroptosis in liver fibrosis

### Ferroptosis in hepatic stellate cells activation

Since the first study reporting that the role of ferroptosis in liver fibrosis (Sui et al., 2018), accumulating evidence has shown that ferroptosis is involved in the pathogenesis of liver fibrosis. Numerous studies have demonstrated that ferroptosis exerts an inhibitory effect on liver fibrosis by inactivating HSCs and inducing HSCs death. Moreover, specific regulators of ferroptosis, including ELAV-like RNA binding protein 1 (ELAVL1) (Zhang et al., 2018), bromodomain-containing protein 7 (BRD7) (Zhang et al., 2020a), ZFP36 ring finger protein (ZFP36) (Zhang et al., 2020b), and tripartite motif-containing protein 26 (TRIM26) (Zhu et al., 2021), have been reported to play crucial roles in regulating ferroptosis in HSCs and function as promising targets in preventing liver fibrosis. ELAVL1, a ubiquitous RNA-binding protein, promoted autophagy activation by binding to BECN1/Beclin1 mRNA, thereby enhancing ferroptosis. Ablation of autophagy completely impaired ELAVL1-mediated ferroptosis, whereas induction of autophagy induced a synergistic effect with ELAVL1. In mice, ELAVL1 expression was upregulated by the ferroptosis inducers sorafenib and erastin by inhibiting the ubiquitin–proteasome pathway. In this process, upregulated ELAVL1 binds to BECN1 mRNA and promotes BECN1/Beclin1 generation, resulting in autophagy-dependent degradation of ferritin, HSCs ferroptosis, and attenuated liver fibrosis. Specific knockdown of ELAVL1 in HSCs impaired sorafenib-induced ferroptosis of HSCs in murine liver fibrosis (Zhang et al., 2018). ZFP36,a RNA-binding protein, can destabilize autophagy related 16 like 1 (ATG16L1) mRNA, thus inhibiting macroautophagy/autophagy activation and mediating ferroptosis resistance. The ferroptosis inducer sorafenib was shown to downregulate ZFP36 protein expression by the ubiquitin ligase F-box and WD repeat domain containing 7 (FBXW7/CDC4). Therefore, erastin and sorafenib treatment can ameliorate liver fibrosis by downregulating ZFP36, activating ferritinophagy, and ferroptosis in HSCs (Zhang et al., 2020a). Ferritinophagy is a type of autophagy mediated by nuclear receptor activator 4 (NCOA4), which plays a role in inducing ferroptosis by regulating iron homeostasis and producing ROS in cells (Gao et al., 2016; Hou et al., 2016; Liu et al., 2022). NCOA4 acts as a selective autophagy receptor and binds to ferritin heavy chain-1 (FTH1) of ferritin to mediate the transport of intracellular ferritin to autophagy lysosomes and finally release free iron (Mancias et al., 2014). NCOA4-mediated ferritinophagy degrade ferritin resulting in a feedback regulation mechanism of available iron in cells, and its activation will increase the content of available iron in cells (He et al., 2022). In mice, treatment with erastin and sorafenib alleviated liver fibrosis by inducing HSCs ferroptosis. HSCs-specific overexpression of *Zfp36* impaired erastin- or sorafenib-induced HSCs ferroptosis, suggesting that ZFP36 may function as a ferroptosis inhibitor. Another study reported the ability of BRD7 to function as ferroptosis inducer targeting HSCs (Zhang et al., 2020b). BRD7 directly binds to promote p53 mitochondrial translocation, which subsequently forms complexes with and elevates the activity of solute carrier family 25 member 28 (SLC25A28), leading to excessive deposition of redox-active iron and HSCs ferroptosis. Genetically blocking the binding of BRD7 to p53 inhibited mitochondrial translocation of p53 and ferroptosis in HSCs. Silencing SLC25A2 impaired BRD7-or p53-induced HSCs ferroptosis. *In vivo*, the ferroptosis inducer erastin abrogated liver fibrosis by inducing HSCs ferroptosis. Moreover, HSCs-specific blockade of BRD7/P53/SLC25A28 axis could ameliorate erastin-induced ferroptosis in HSCs. It was shown that erastin and sorafenib can suppress murine liver fibrosis by inducing HSCs ferroptosis *via* the BRD7/P53/SLC25A28 axis (Zhang et al., 2020a). Moreover, sorafenib was shown to attenuate liver fibrosis by triggering HSCs ferroptosis *via* hypoxia-inducible factor (HIF)-1α/SLC7A11 signaling in a carbon tetrachloride (CCl_4_)-induced mouse liver fibrosis model (Yuan et al., 2022). A recent study also demonstrated that TRIM26, an E3 ubiquitin ligament, was downregulated in fibrotic liver tissues. TRIM26 overexpression promoted HSCs ferroptosis by mediating the ubiquitination and degradation of SLC7A11, thereby inhibiting HSCs proliferation (Zhu et al., 2021). Another recent study demonstrated that N6-methyladenosine (m^6^A) modification enhances HSCs ferroptosis while methylase METTL4 and demethylase FTO competitively stabilized YTHDF1-dependant BECN1 mRNA, thereby activating autophagy and eventually resulting in HSCs ferroptosis (Shen et al., 2021). Taken together, these findings suggest that the pharmacological induction of HSCs ferroptosis may represent a therapeutic target for novel liver fibrosis treatment strategies.

### Hepatocyte ferroptosis

Few studies have reported on the role of hepatocyte ferroptosis in the development of liver fibrosis. The evidence suggests that hepatocyte and HSCs ferroptosis exerts opposite effects. High iron overload, serving as a driving factor in ferroptosis induction, increased the risk of developing liver fibrosis and cirrhosis. Such excessive iron accumulation enhanced ferroptosis in hepatocytes by inducing heme oxygenase 1 (HO-1) expression, which contributed to the progression of liver fibrosis, accompanied by the upregulation of the FGF21 protein level in *vitro* and *in vivo* (Wu et al., 2021). During ferroptosis, HO-1 may play a pro-death role in enhancing iron release, thereby being implicated in the initiation of ferroptosis (Ryter, 2021).

In a db/db mouse model of T2DM, increased TGF-β, collagen I, and collagen III levels in hepatic cells indicated greater liver fibrogenesis (Song et al., 2022). Ferroptosis activation was observed in hepatic cells, showing that increased ROS production; SOD, GSH-PX, and GSH downregulation; and MDA, 4-HNE, and NOX4 upregulation increased TfR1 expression, decreased FPN1 expression, and downregulated SLC7A11 and the Nrf2/HO-1/GPX4 signaling pathway (Song et al., 2022). The anti-obesity and diabetes drug liraglutide was also shown to alleviate liver fibrosis by inhibiting ferroptosis (Song et al., 2022). Taken together, these findings suggest that the induction of hepatocyte ferroptosis may be involved in the pathogenesis of T2DM-related liver fibrosis. However, the precise function of hepatocyte ferroptosis in the development and pathogenesis of liver fibrosis remains poorly understood. The molecular mechanism by which hepatocyte ferroptosis is regulated remains largely unknown and warrants further investigation.

## Pharmacological modulation of ferroptosis to treat liver fibrosis

### Pharmacological induction of HSCs ferroptosis as a therapeutic target for liver fibrosis

**FIGURE 3:**
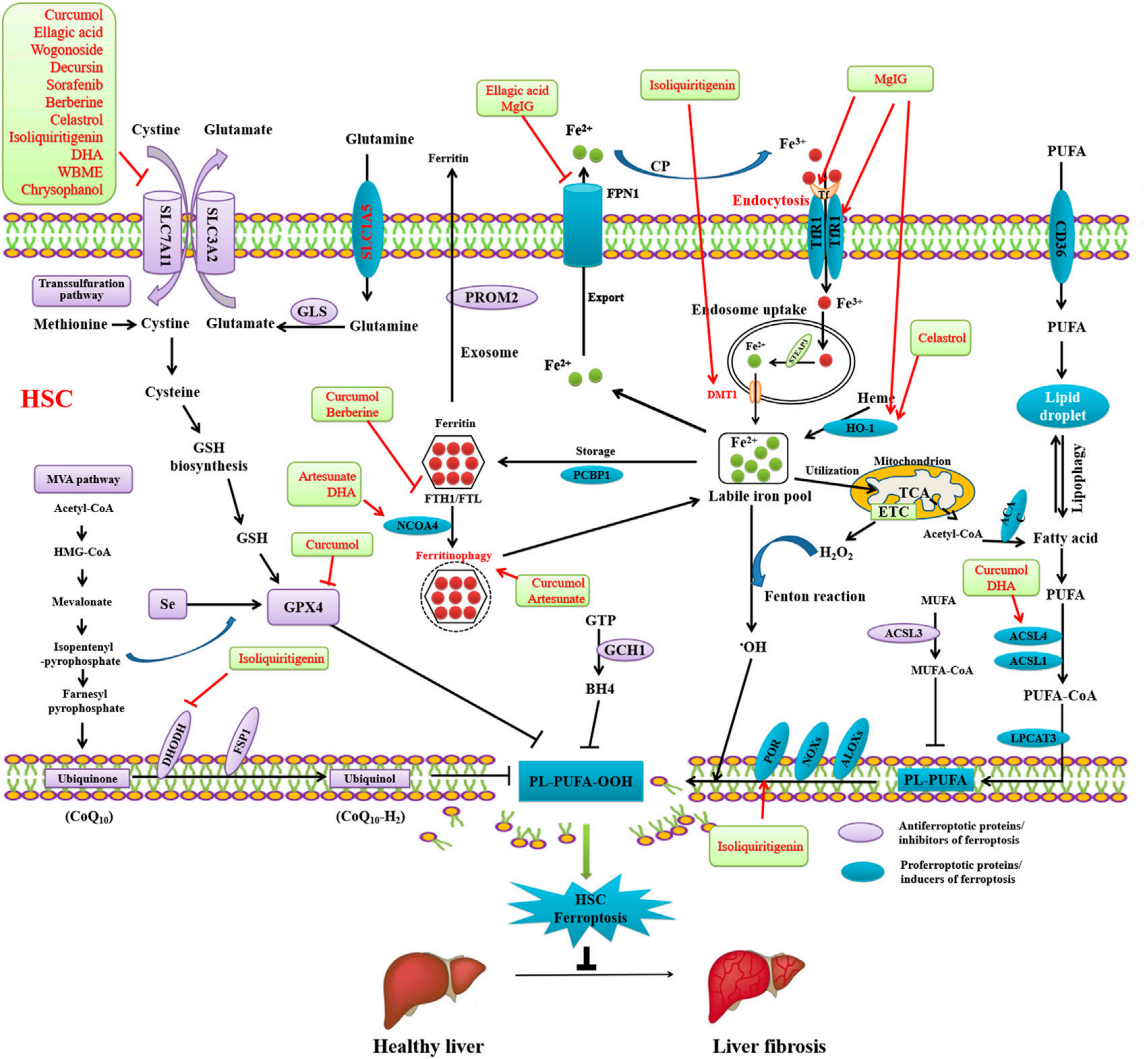
Reversing liver fibrosis through inducing ferroptosis by bioactivative compounds in HSCs. Curcumol, ellagic acid, wogonoside, decursin, sorafenib, berberine, celastrol, isoliquiritigenin, DHA, WBME, and chrysophanol alleviate liver fibrosis through inducing ferroptosis by inhibiting SLC7A11. Curcumol inhibits GPX4, thereby inducing HSCs ferroptosis to alleviate liver fibrosis. Isoliquiritigenin inhibits DHODH and upregulates POR, thereby inducing ferroptosis to alleviate liver fibrosis. Artesunate and DHA induces NCOA4-mediated ferritinophagy to induce ferroptosis and alleviate liver fibrosis. Curcumol and DHA induced ACSL4-dependant ferroptosis to alleviate liver fibrosis. Berberine and curcumol alleviate liver fibrosis through inhibiting FTH1/FTL. Ellagic acid and MgIG downregulates FPN1 to induce ferroptosis and alleviate liver fibrosis. MgIG upregulates TfR1 and HO-1 to induce ferroptosis and alleviate liver fibrosis. Celastrol also upregulates HO-1 to induce ferroptosis and alleviate liver fibrosis.

Recent studies have demonstrated that the pharmacological scavenging of HSCs by activating ferroptosis has potential as a novel therapeutic strategy for liver fibrosis (Figure 3). Mounting evidence from *in vitro* and *in vivo* models indicates that pharmacological induction of ferroptosis may mitigate liver fibrosis development (Table 1). Moreover, there is an increasing body of evidence showing that inducing HSCs ferroptosis using Curcumol (Zheng et al., 2022), ellagic acid (Li et al., 2022), phloridzin (Shi et al., 2022), liraglutide (Song et al., 2022), berberine (Yi et al., 2021), wogonoside (Liu et al., 2022), decursin (Que et al., 2022), celastrol (Luo et al., 2022),isoliquiritigenin (Huang et al., 2022), DHA (Zhang et al., 2021; Shen et al., 2022), wild bitter melon extract (WBME) (Ho et al., 2021),chrysophanol (Kuo et al., 2020), magnesium isoglycyrrhizinate (Sui et al., 2018), artesunate (Kong et al., 2019), and artemether (Wang et al., 2019) has potential as a therapeutic strategy to prevent or inhibit liver fibrosis. Curcumol (Zheng et al., 2022), ellagic acid (Li et al., 2022), wogonoside (Liu et al., 2022), decursin, sorafenib, berberine, celastrol (Luo et al., 2022), isoliquiritigenin (Huang et al., 2022), DHA (Zhang et al., 2021), WBME (Ho et al., 2021), and chrysophanol (Kuo et al., 2020) alleviate liver fibrosis through inducing ferroptosis by inhibiting SLC7A11. Curcumol inhibits GPX4, thereby inducing HSCs ferroptosis to alleviate liver fibrosis (Zheng et al., 2022). Isoliquiritigenin inhibits DHODH and upregulates POR, thereby inducing ferroptosis to alleviate liver fibrosis (Sui et al., 2018). Artesunate (Kong et al., 2019) and DHA (Zhang et al., 2021) induces NCOA4-mediated ferritinophagy to induce ferroptosis and alleviate liver fibrosis. Curcumol (Zheng et al., 2022) and DHA (Zhang et al., 2021) induced ACSL4-dependant ferroptosis to alleviate liver fibrosis. Berberine (Yi et al., 2021) and curcumol (Zheng et al., 2022) alleviate liver fibrosis through inhibiting FTH1/FTL. Ellagic acid (Li et al., 2022) and MgIG (Sui et al., 2018) downregulates FPN1 to induce ferroptosis and alleviate liver fibrosis. MgIG (Sui et al., 2018) upregulates TfR1 and HO-1 to induce ferroptosis and alleviate liver fibrosis (Sui et al., 2018). Celastrol also upregulates HO-1 to induce ferroptosis and alleviate liver fibrosis (Luo et al., 2022).

**TABLE 1:** Compounds alleviate liver fibrosis through inducing ferroptosis in HSCs.

Drugs	Experimental model	Effects	Mode of action	Ref
Curcumol	HSC-T6 cells	↑Death of activated HSCs; ↓deposition of extracellular matrix; ↑Autophagy; ↓SLC7A11 and GPX4; ↑ ACSL4 and COX2; ↑MDA; ↑ROS; ↓GSH; ↑Fe^2+^;↑NCOA4; ↓ FTH1	↑HSCs ferroptosis	Zheng et al. (2022)
Ellagic acid	CCl_4_-induced Male C57BL/6 mice	↓ALT, AST and LDH activities; ↓ PC-III, LN, and collagen in serum; ↓degree of collagen deposition in mouse livers; ↓α-SMA, collagen type 1 (Col1a1) and TGF-β1; ↓desmin; ↓α-SMA, Col1a1 and Tgf-β1 mRNA; ↓protein levels of α-SMA and TGF-β1; ↑ FTH1 content (a main iron storage protein); impairs the formation of VAMP2/syntaxin 4 and VAMP2/synaptosome-associated protein 23 complexes by suppressing VAMP2 expression by enhancing its degradation in a proteasome-dependent pathway. This leads to the impairment of ferroportin (FPN, an iron exporter) translocation and intracellular iron extrusion	↑HSCs ferroptosis	Li et al. (2022)
Ellagic acid	Bile duct ligation (BDL) mouse mode	↓ liver fibrosis;↓ α-SMA and TGF-β1 gene expression was verified by the results of immunohistochemistry, western blot and qPCR assays	↑HSCs ferroptosis	Li et al. (2022)
Ellagic acid	Bile duct ligation (BDL) mouse mode	↓ liver fibrosis; ↓ α-SMA and TGF-β1 gene expression was verified by the results of immunohistochemistry, western blot and qPCR assays	↑HSCs ferroptosis	Li et al. (2022)
Ellagic acid	TGF-β1-induced human LX-2 cells	↑ growth inhibition; ↓ α-SMA, col1a1 and desmin expression; ↓ matrix metalloproteinase (MMP)-2 and MMP-9; ↑GSH depletion; ↑ redox-active iron accumulation; ↑ lipid peroxidation; ↓FPN	↑HSCs ferroptosis	Li et al. (2022)
Ellagic acid	TGF-β1-induced primary mouse HSCs	↑ growth inhibition; ↓ α-SMA, col1a1 and desmin expression; ↓ matrix metalloproteinase (MMP)-2 and MMP-9; ↑GSH depletion; ↑ redox-active iron accumulation; ↑ lipid peroxidation; ↓FPN	↑HSCs ferroptosis	Li et al. (2022)
Ellagic acid	CCl_4_-induced primary mouse hepatocyte	Not suppress the growth and viability of the hepatocytes; did not trigger GSH depletion, redox-active iron overload or MDA production in primary hepatocytes or HepG2 cells	No effects	Li et al. (2022)
Phloridzin	CCl_4_-induced C57/BL6N mouse	↓Collagen deposition and decreased levels of serum ALT, AST, laminin, and HA	NA	Shi et al. (2022)
Berberine	TAA and CCl_4_ induced mouse	↓Liver fibrosis; ↑liver function; ↓Autophagy of HSCs; ↓HSCs activation *in vivo* and *in vitro*; ↑ROS in HSCs; ↑HSCs ferroptosis; ↑Ptgs2; ↓SLC7A11 and GPX4; ↓FTH11 and FTL; ↑p62,LC3-II,LC3-II	↑HSCs ferroptosis	Yi et al. (2021)
Sorafenib	CCl_4_-induced C57/BL6N mouse	↓Liver injury and ECM accumulation; ↓SLC7A11 and GPX4 of HSCs	↑HSCs ferroptosis	Yuan et al. (2022)
Sorafenib	Sorafenib treated HSC-T6 cells	↓SLC7A11, GPX4 and GSH; ↑accumulation iron, ROS and MDA; ↓HIF-1α and SLC7A11	↑HSCs ferroptosis	Yuan et al. (2022)
Wogonoside	CCl_4_ induced mouse	↓Liver fibrosis (α-SMA,COL1α1); ↑SOCS1 and P53	↑SOCS1/P53	Liu et al. (2022a)
Wogonoside	CCl_4_ induced HSC-T6 cells	↓Cell viability; ↓ECM expression; ↓SLC7A11, GPX4 and GSH; ↑iron, ROS and MDA; ↑SOCS1 and P53	↑HSCs ferroptosis	Liu et al. (2022b)
Decursin	CCl_4_ induced C57BL/6 J mice	↓Liver fibrosis (collagen, α-SMA, and Col1α1); ↑Fe^2+^, lipid ROS; ↓ GPX4 and GSH in HSCs	↑HSCs ferroptosis	Que et al. (2022)
Decursin	Primary HSCs from CCl_4_ induced C57BL/6 J mice	↓GPX4 and GSH; ↑ Fe^2+^, ROS, and Ptgs2	↑HSCs ferroptosis	Que et al. (2022)
Decursin	TGF-β-activated LX-2 cells	↓Cell viability; ↑Ptgs2, Fe^2+^, ROS, and MDA; ↓ GPX4 level and GSH	↑HSCs ferroptosis	Que et al. (2022)
Celastrol	CCl_4_ induced C57BL/6 male mice	↓Liver injury (serum levels of AST, ALT and ALP); ↓Liver fibrosis (collagen, α-SMA, and Col1α1); ↓GPX4; ↑COX2; ↓anti-oxidant activities of PRDXs	↑GPX4; ↓LPO	Luo et al. (2022)
Celastrol	LX-2 cells	↑ Fe^2+^/Fe^3+^ and LPO; ↓ GSH; ↑ROS; ↑HO-1	↑HSCs ferroptosis	Luo et al. (2022)
Isoliquiritigenin	CCl_4_ induced C57BL/6 mice	↓Liver injury (serum levels of AST and ALT); ↑DMT1	-	Huang et al. (2022)
Isoliquiritigenin	HSC-T6 cells	↑Cells viability; ↓Liver injury; ↑ROS; ↑TFR; ↑COX2; ↑DMT1; ↑POR; ↓SLC7A11; ↓GPX4; ↓DHODH	↑HSCs ferroptosis	Huang et al. (2022)
Isoliquiritigenin	TAA induced zebrafish	↓Liver fibrosis (collagen, α-SMA, and Col1α1); ↑iron; ↑ROS	-	Huang et al. (2022)
DHA	CCl_4_ induced SD rat	↓Liver injury (serum ALT, AST, ALP, LDH and TBIL); ↓Liver fibrosis (α-SMA, collagen I, fibronectin); ↓collagen deposition	↑HSCs ferroptosis	Zhang et al. (2021)
DHA	PDGF-BB-induced primary HSCs	↓Cells viability; ↓Liver fibrosis; ↓α-SMA, collagen I, fibronectin and MMP13 (protein and mRNA); ↓collagen deposition; ↓TGF-βR and PDGF-Rβ mRNA; ↑ACSL4; ↓SLC7A11; ↑ Fe^2+^, ROS, and MDA; ↓ GPX4 and GSH; ↑NCOA4	↑HSCs ferroptosis	Zhang et al. (2021)
MgIG	CCl_4_ induced SD rat	↓Liver injury (serum ALT, AST, ALP, LDH and TBIL); ↓serum HA, LN, PC-III and IV-C levels; ↓Liver fibrosis (α-SMA, collagen I, fibronectin and demin); ↓TGF-βR1 and PDGF-βR	-	Sui et al. (2018)
MgIG	HSC-T6 cells	↓α-SMA, collagen1, demin and fibronectin; ↓TGF-βR1,PDGF-βR and EGFR; ↓TIMP1 and TIMP2; ↑MMP2 and MMP9; ↑ Fe^2+^, ROS, 4-HNE and MDA; ↑TF,TfR,FTH1, HO-1; ↓FPN	↑HSCs ferroptosis	Sui et al. (2018)
Artesunate	Primary HSCs isolated from CCl_4_ induced mice	↓Cell vitality; ↑cell death rate; ↑iron; ↑lipid peroxides and reduced antioxidant capacity; ↑LC3,Atg3, Atg5, Atg6/beclin1, Atg12; ↓p62, FTH1, NCOA4; ↑ferritinophagy	↑HSCs ferroptosis	Kong et al. (2019)
Artesunate	CCl_4_ induced mice	↑HSCs ferroptosis; ↓Liver fibrosis including HA, LN, PC-III, and IVeC; ↑Fe^2+^, lipid ROS, Ptgs2 mRNA; ↓GSH	↑HSCs ferroptosis	Kong et al. (2019)
Artemether	CCl_4_ induced mice	↓Liver fibrosis; ↓α-SMA, collagen I, fibronectin (protein and mRNA); ↓Liver injury (ALT, AST and ALP levels in liver and serum); ↓serum HA, LN, PC-III and IV-C levels; ↓ECM deposition (massive collagen); ↓hydroxyproline in liver and serum; ↓PDGF-βR and EGFR	↑HSCs ferroptosis	Wang et al. (2019)
Artemether	HSC-T6 cells	↓HSCs activation (↓α-SMA, α1(I)collagen, and fibronectin); ↑ Fe^2+^ and LPO; ↓TGF-βR1, PDGF-βR, and EGFR (IF); α-SMA, α1(I)collagen, fibronectin, TGF-βR1, PDGF-βR, and EGFR (WB); α-SMA, α1(I) collagen and fibronectin (RT-PCR)	↑HSCs ferroptosis	Wang et al. (2019)
WBME	LPS-induced HSCs-T6 cells	↑Cell death rate; ↑ROS; ↑ lipid ROS; ↓CTGF,α-SMA,integrin-β1 and p-JNK; ↓ GPX4 and SLC7A11; ↑CHOP	↑HSCs ferroptosis	Ho et al. (2021)
Chrysophanol	Rat HSC-T6 cells expressing HBx	↓Cell vitality; ↑ lipid ROS; ↑ER stress; ↓CTGF,α-SMA; ↓ GPX4 and SLC7A11	↑HSCs ferroptosis	Kuo et al. (2020)
DHA	HSC-LX2 and Primary	↓HSCs activation; ↓α-SMA, fibronectin and collagen I; ↑ Fe^2+^;↑ ROS; ↑ MDA;↓ GSH; ↑ autophagy; ↑ FTO-mediated m^6^A methylation of BECN1	↑HSCs ferroptosis	Shen et al. (2022)
DHA	CCl_4_ induced mice	↓Liver injury; ↓Liver fibrosis; ↓α-SMA (ICH); ↓ACTA2, collagen I and demin (mRNA)	↑HSCs ferroptosis	Shen et al. (2022)

↑,promote, upregulate or increase; ↓,inhibit, downregulate or decrease; α-SMA, α-smooth muscle actin; ACSL4, acyl-coA synthetase long-chain family member four; AST, aspartate aminotransferase; ALT, alanine aminotransferase; ALP, alkaline phosphatase; Atg, autophagy related genes; CHOP, CCAAT enhancer-binding protein homologous protein; CCl_4_, carbon tetrachloride; COL1α1,α1(I)collagen; CTGF, connective tissue growth factor; DHA, dihydroartemisinin; ECM, extracellular matrix; FGF21, Fibroblast growth factor 21; GPX4, glutathione peroxidase 4; GSH, glutathione; HA, hyaluronic acid; HSCs, hepatic stellate cell; HYP, hydroxyproline; IV C, collagen type IV; LC3, microtubule-associated protein light chain 3; PDGF-BB, Platelet-derived growth factor BB; FPN, ferroportin; FTH1, ferritin heavy chain 1; LDH, lactate dehydrogenase; LPS, lipopolysaccharid; MDA, malondialdehyde; MgIG, magnesium isoglycyrrhizinate; MMP13, matrix metalloprotease 13; NCOA4, nuclear receptor co-activator 4; PC-III, procollagen type III; PDGF, platelet-derived growth factor; PDGF-BB, platelet-derived growth factor BB; Ptgs2,Prostaglandin endoperoxide synthase 2; ROS, reactive oxygen species; SLC7A11, solute carrier family 7 member 11; TBIL, total bilirubin; TF, transferrin; TGFβ, transforming growth factor β; TfR, transferrin receptor; TAA, thioacetamide; WBME, Wild Bitter Melon Extract; VAMP2,vesicle-associated membrane protein 2.

### Pharmacological inhibition of hepatocyte ferroptosis as a therapeutic target for liver fibrosis

To the best of our knowledge, there have been few reports on the pharmacological inhibition of hepatocyte ferroptosis for treating liver fibrosis (Wu et al., 2021) (Table 2). Recombinant FGF21 significantly protected against liver fibrosis by inhibiting hepatocyte ferroptosis (Wu et al., 2021). FGF21 ablation aggravated iron overload-induced hepatocyte ferroptosis, and FGF21 inhibited of HO-1 expression through promoting its ubiquitination and degradation. These findings indicate that FGF21 functions as a novel ferroptosis suppressor, and its activation in hepatocytes may serve as a potential therapeutic target for liver fibrosis. A recent study demonstrated that liraglutide can alleviate liver fibrosis by inhibiting hepatocyte ferroptosis through upregulating SLC7A11 expression and the Nrf2/HO-1/GPX4 pathway (Song et al., 2022). Liraglutide improved liver function and suppressed liver fibrosis in db/db mice; moreover, it inhibited ROS production and LPO by upregulating SOD, GSH-PX, and GSH activity and downregulating MDA, 4-HNE, and NOX4 expression (Song et al., 2022). Liraglutide was also found to attenuate iron accumulation by decreasing TfR1 expression and increasing FPN1 expression (Song et al., 2022). Together, these results show that liraglutide has potential for inhibiting T2DM-related liver fibrosis by limiting hepatocyte ferroptosis.

**TABLE 2:** Compounds alleviate liver fibrosis through inhibiting ferroptosis in hepatocytes.

Drugs	Experimental model	Effects	Mode of action	Ref
Liraglutide	db/db mice	↓Liver fibrosis; ↓Serum AST and ALT levels↓ROS; ↑SOD, GSH-PX, and GSH activity; ↓MDA, 4-HNE, and NOX4; ↓Fe^2+^;↓TfR1; ↑FPN1; ↑SLC7A11; ↑Nrf2/HO-1/GPX4	↓Hepatocytes ferroptosis	Song et al. (2022)
FGF21	Iron overload-induced hepatocytes	↓Liver injury and fibrosis by inhibiting hepatocytes ferroptosis	↓Hepatocytes ferroptosis	Wu et al. (2021a)

## Conclusion and perspectives

This review summarizes the recent progress of research on the pathological pathways and regulation mechanisms of ferroptosis in liver fibrosis and reviews the application of ferroptosis modulators to mitigate liver fibrosis. Moreover, new targets were identified for future treatment and prevention of liver fibrosis through targeting ferroptosis. Emerging evidence have suggested that pharmacological induction of HSCs ferroptosis or inhibition of hepatocytes ferroptosis both have potential as therapeutic targets for managing or inhibiting liver fibrosis, which elucidates possibilities for the discovery of novel targets and drug strategies for liver fibrosis. However, the current literature on the role of ferroptosis in liver fibrosis remains somewhat limited, and more studies are required to clarify its role and functional mechanisms. Relatively little is known about how ferroptosis orchestrates diverse cellular events. Firstly, the regulatory mechanism underlying the ferroptosis of hepatocytes in liver fibrosis must be identified. Secondly, ferroptosis of different cell types may exert widely variable effects, and strategies aiming to induce ferroptosis in HSCs to inhibit liber fibrosis may aggravate fibrosis by inducing ferroptosis in hepatocytes owing to these differential effects. Therefore, ferroptosis-targeted therapies may have unpredictable effects in some patients with liver fibrosis and lead to side effects. Considering this, ferroptosis-targeted therapies require careful consideration. Possible side effects may be largely alleviated by the development of cell type–targeting drug delivery systems. At present, possible unwarranted toxic adverse events, poor drug targeting, and limited treatment effect among others are the major problems the anti-liver fibrosis drugs should to overcome (Poelstra and Schuppan, 2011; Peng et al., 2021). Current treatment strategies for liver fibrosis are directly targeting to eliminate related injury factors, inhibit the activation of HSCs, increase the degradation of ECM and decrease inflammatory reactions (Ebrahimi et al., 2018; Peng et al., 2021). HSCs are the primary effector cells in liver fibrosis, and most drugs are required to target HSC to treat liver fibrosis. And how to target HSCs has been the main focus of anti-liver fibrosis research in recent years. The nano-drug delivery system is a new and safe drug delivery system with many advantages are now widely used for the treatment of liver fibrosis (Zhou et al., 2022). Numerous nanocarriers have been designed to combat liver fibrosis through modifying the surface receptors of HSCs or specific ligands of highly expressed receptors on the surface of inorganic nanoparticles, liposomes, protein polymers, and other nanodrug delivery systems such as HSCs surface receptors or high expression of receptor specificity ligand (Peng et al., 2021). Targeting nanocarriers to specific liver cell types have the potential to suppress off-target and adverse effects, thereby increasing drugs efficacy (Kumar et al., 2021). Hepatocytes are the primary target of lipotoxicity and oxidative stress in NAFLD and NAFLD-associated liver fibrosis (Kumar et al., 2021). So targeting inhibition of ferroptosis through nanocarriers maybe a ideal strategies to treat NAFLD-associated liver fibrosis with few side effects. Together, cell-specificity reached by cell-specific drug carriers according to different causes that result in liver fibrosis is essential for most anti-liver fibrosis drugs. The future research should continues to develop smart nanocarriers to facilitate a personalized medicine for liver fibrosis. Furthermore, most data reported in the literature are derived from experimental studies that do not directly report clinical applications and implications. Hence, more clinical studies must be conducted to inform practical treatment and management strategies. Despite these considerations, the current evidence strongly indicates that targeting ferroptosis marks a significant new direction for treating liver fibrosis.
